# Extraction and Chemical Characterization of Functional Phenols and Proteins from Coffee (*Coffea arabica*) By-Products

**DOI:** 10.3390/biom11111571

**Published:** 2021-10-22

**Authors:** Barbara Prandi, Maura Ferri, Stefania Monari, Chiara Zurlini, Ilaria Cigognini, Stefanie Verstringe, Dennis Schaller, Martha Walter, Luciano Navarini, Annalisa Tassoni, Stefano Sforza, Tullia Tedeschi

**Affiliations:** 1 Department of Food and Drug, University of Parma, Parco Area delle Scienze 17/A, 43124 Parma, Italy; barbara.prandi@unipr.it (B.P.); stefano.sforza@unipr.it (S.S.); 2 Department of Civil, Chemical Environmental and Materials Engineering, University of Bologna, Viale del Risorgimento 2, 40136 Bologna, Italy; maura.ferri@unibo.it; 3 Department of Biological, Geological and Environmental Sciences, University of Bologna, Piazza di Porta S. Donato 1, 40127 Bologna, Italy; stefania.monari2@unibo.it (S.M.); annalisa.tassoni2@unibo.it (A.T.); 4 Stazione Sperimentale per l’Industria delle Conserve Alimentari, Viale Tanara 31/A, 43121 Parma, Italy; chiara.zurlini@ssica.it (C.Z.); ilaria.cigognini@ssica.it (I.C.); 5 Nutrition Sciences N.V., Booiebos 5, 9031 Drongen, Belgium; s.verstringe@agrifirm.com; 6 IGV GmbH, Arthur-Scheunert-Allee 40-41, 14558 Nuthetal, Germany; dennis.schaller@igv-gmbh.de (D.S.); martha.walter@igv-gmbh.de (M.W.); 7 Illycaffè S.p.A, Via Flavia 110, 34147 Trieste, Italy; luciano.navarini@illy.com

**Keywords:** coffee, agri-food, by-products, proteins, polyphenols, enzymes

## Abstract

Not all the coffee produced goes to the roasting stage, because non-compliant green coffee beans are usually discarded by roasters and the silverskin of the coffee is usually removed and discarded. In the present work, non-compliant green coffee beans and coffee silverskins were fully characterized from a chemical point of view. In addition, enzyme-assisted extraction was applied to recover a fraction rich in proteins and polyphenols, tested for antimicrobial, antityrosinase, and antioxidant activities. Non-compliant green coffee beans showed higher amounts of polyphenols, flavanols, flavonoids, and caffeine than coffee silverskins (which were richer in tannins). The enzymatic extraction of non-compliant coffee green beans produced extracts with a good protein content and with a consistent quantity of polyphenols. The extract showed antioxidant, antityrosinase, and antimicrobial activity, thus representing a promising strategy to recover defective green coffee beans. The antioxidant and antimicrobial activity of coffee silver skins is lower than that of non-compliant coffee green beans extracts, while the antityrosinase activity is comparable.

## 1. Introduction

Global coffee production reached 170.94 million 60-kg bags in 2019, primarily produced in South America, and in Brazil (61.7 million 60-kg bags), followed by Vietnam and Colombia. The leading importer is the United States ($5.7 billion), followed by Germany and France ($3.3 and $2.8 billion) [1]. The two main species used to produce coffee are *Coffea canephora* (commonly called “Robusta”) and *Coffea arabica* (“Arabica”). Most of the world coffee production is made up of Arabica (69–74%), with the remaining 25–30% made up of Robusta [2].

Roasters select coffee green beans (CGBs) to eliminate those that do not meet the required quality standards (non-compliant). The quantity of CGBs discarded depends on the selection criteria of the suppliers and the quality standards applied. For example, these defective beans make up about 20% of total coffee production in Brazil and are separated from non-defective beans before being marketed [3]. Then, the green beans are processed, using a dry or wet method. Two types of wastes are produced during wet processing: coffee pulp (29% of the initial weight) and coffee parchment husk (12%). During dry processing, approximately 12% of the initial raw product is lost in removing the cherry husk from the coffee. Finally, the processed green seeds are roasted, and during this step, the coffee silver skin (CSS, 1%) is removed. The environmental impact of CSS is given by the combination of several factors, such as the considerable production volume, their flammability, and the energy required in the compaction phase for their disposal [4]. In addition to the by-products of the coffee industry, a significant amount of coffee waste (45%) is generated during the preparation of the beverage, [5] at home or at hotellerie-restaurant-café (HORECA). The composition of all these by-products shows interesting potential, with many being a rich source of valuable compounds, which could be recovered for further enhancement and valorization.

Green seeds of Arabica coffee contain 46–53% insoluble polysaccharides (cellulose, hemicellulose, and β-1,4-mannan), 8.5–12% proteins, 15–18% lipids, and 3–4.5% minerals [6]. CGBs and their press cake (remaining after the extraction of the oil) have in fact been studied for the recovery of bioactive compounds, specifically polyphenols with antioxidant activity. In this case, the antioxidant capacity of the by-product was even higher than that of the green beans [7].

CSS contains 50–60% dietary fiber, 16–19% protein, 1.6–3.3% fat, and about 7% ash [8]. The CSS extract can therefore be considered a source of protein, fiber (including soluble dietary fiber [9]), and minerals (K^+^, Mg^2+^, and Ca^2+^), with a low fat content [10]. Additionally, CSSs are also a rich source of polyphenols [11]. Consequently, the antioxidant fraction of CSS has already been studied for its antioxidant activity, and for possible inclusion in skin care products [12]. CSS extracts (composed mainly of caffeine and chlorogenic acid [13]) have also been studied for their properties to reduce adipogenesis and inflammation-related ailments [14]. The protein fraction of CSS has also been evaluated for its biofunctional properties: the peptides deriving from protease treatment have been studied for their antioxidant or hypocholesterolemic activity [15]. The packaging sector has also shown interest in CSSs, which have in fact been successfully inserted into poly(lactic acid) and poly(butylene succinate) polymers by up to 30% by weight [16]. A recent study has shown that CSS can be incorporated into a poly(3-hydroxybutyrate-co-3-hydroxyvalerate) (PHBV) matrix, producing highly efficient PHBV/CS-based biocomposites [17].

Overall, the recovery of coffee by-products has focused, up to now, mainly on the polyphenol and fibrous components. At the same time, protein content and further valorization have been much less studied. As mentioned above, peptides derived from the protein fraction could have interesting biofunctional properties. These peptides could also be synergized with polyphenols to obtain extracts with improved properties.

A mild environmentally friendly approach for protein recovery, still little studied on coffee by-products, could use proteases to obtain a simultaneous extraction and hydrolysis of proteins from coffee by-products, possibly with simultaneous extraction of the polyphenols present. This approach would be a rather innovative application on coffee byproducts, as up to now, only enzymes that act on the carbohydrate fraction have been used to hydrolyze spent coffee ground [18]. Only one study evaluated the use of protease to simultaneously extract oil and proteins from green coffee [19].

Therefore, the aim of the present work was to apply for the first time a protease-assisted extraction to recover the protein fraction of the two most important by-products of the coffee industry, the defective CGB and the CSS. With this approach, peptide/polyphenol mixtures were obtained, which were characterized by their composition. As the target applications of these extracts are their possible use as antioxidant compounds in active food packaging films, as anti-aging compounds to be included in cosmetic formulations, and as a feed additive with antimicrobial properties, their antioxidant, anti-tyrosinase, and antimicrobial properties were also tested. The possible correlation between the observed biofunctional properties and the composition of the extracts will be discussed, with the general aim of exploring enzyme-assisted extraction as a possible way of valorizing coffee by-products.

## 2. Materials and Methods

**Figure 1:**
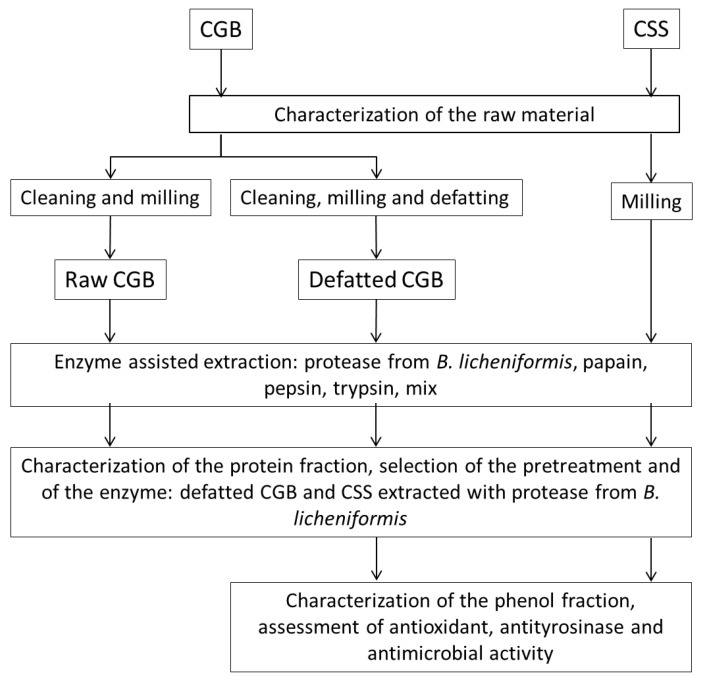
Experimental scheme showing the main phases of the research.

The general objective of the work was to characterize two coffee by-products (CGB and CSS), to evaluate the enzyme-assisted extraction technique for the simultaneous recovery of proteins and polyphenols and to evaluate some bioactive properties of the selected extracts. The experimental scheme of the present work is shown in Figure 1. Then, the two by-products were characterized with centesimal analysis and characterization of the protein and phenolic fractions. In the case of CGB, two different pre-treatments were also compared, while for CSS, only coarse grinding was necessary. Then, enzyme-assisted extraction was applied to co-extract proteins and polyphenols, comparing the efficiency of four different enzymes. The protein fraction of the resulting extracts was characterized, and the two selected extracts were also characterized for their phenolic content and different bioactivities.

### 2.1. Sampling of Raw Materials

The raw materials were selected among the most interesting from the point of view of industrial waste management, and for the possible contents of proteins and bioactive molecules. Non-compliant green coffee (*Coffea arabica* L.) beans, n-CGBs, from industrial selection processing (illycaffè S.p.A. Trieste, Italy), were obtained by subjecting coffee lots from Central and South America, Asia, and Eastern Africa to electronic sorting by means of color mapping as described in [20]. Coffee (*C. arabica* L.) silverskin, CSS, from the industrial roasting process, was supplied by illycaffè S.p.A. (Trieste, Italy) in flakes of sizes from 2 to 5 mm with a bulk density of 0.18 g/cm^3^ and a particle density of 0.71 g/cm^3^. n-CGBs were sampled in September 2018, while CSSs were collected in October 2018.

### 2.2. Proximal Analysis of Raw Materials

A proximal analysis was performed in triplicate (*n* = 3) to determine the macronutrient composition of the selected food by-products. Proximal analysis was performed as described in [21]. Briefly, the total solids were determined gravimetrically by drying the product at 70 °C at reduced pressure (700–760 mmHg), or at 100 °C at atmospheric pressure, for 4 h. The determination of the protein content was based on the Kjeldhal method. The total fiber content of the by-products was determined by using an enzymatic assay, based on the method published in the 16th edition of the Official Methods of Analysis of the Association of Official Analytical Chemists (AOAC). Regarding sugars, the sucrose, D-glucose, and D-fructose content was always determined based on a UV method first published by Boehringer Mannheim GmbH, with a commercial kit from R-Biopharm. As for lipids, their content was determined using the Soxhlet method, published in the 16th edition of the official methods of analysis of the Association of Official Analytical Chemists.

### 2.3. Phenolic Compounds and Caffeine Content of Raw Materials

The phenolic compound and caffeine contents were determined in the raw materials and extracts obtained to understand their composition and to investigate potential bioactivities. The by-products, CGB and CSS, were dried at 80 °C in an oven up to 48 h and ground with an Ultra-Turrax blender (IKA, Kassel, Germany). Aliquots of dried samples (0.40 g dry wight (DW) CSS and 0.46 g DW CGB, corresponding to 0.5 g (fresh weight) FW) were treated with in 5 mL of 95% (*v*/*v*) methanol by overnight shaking at room temperature and subsequently centrifuged at 5000 rpm for 10 min at room temperature. Obtained methanolic samples were used to assess total polyphenol [22] (results are expressed as mg of gallic acid equivalents per g DW, mg gallic acid (GA) eq/g DW), flavonoid [23] (as mg of catechin equivalents per g of DW, mg catechin (CAT) eq/g DW), flavanol [24] (as mg CAT eq/g DW), and tannin contents [25] (as mg/g DW). Total polyphenol content was also determined in the protein extracts, starting from 200 mg/mL extract resuspension in MilliQ water (Merck KGaA, Darmstadt, Germany).

Caffeine was quantified by a spectrophotometric method as in [26], in solid by-products aliquots extracted in boiling water (0.4 g FW CGB or 0.8 g FW CSS, in 40 mL). The results are expressed as mg of caffeine per g of DW (mg CAF eq/g DW).

All assay procedures were performed in duplicate in two technical replicates each. The results are expressed as the mean (*n* = 2) ± SD.

### 2.4. Pretreatments of CGB

The pre-treatment of green coffee beans was necessary to improve the extraction yield and the hygienic conditions of the process.

Cleaning. In the first phase, the defective grains were eliminated. Light parts, such as fragments, small beans, or hollow beans, were removed through an air flow. The sample was checked for further foreign bodies by visual inspection, in which the beans flowed individually on a vibrating channel. Before the sample was placed in the mill and ground, the beans passed through a magnetic separator. To reduce adhering substances, the surface of the CGB was mechanically processed. Existing deposits and parts of the shell were removed by means of a counter-current mixer. This abrasion was then removed by means of an air flow. This cleaning improved the hygienic conditions of the samples.

Milling. The grinding was carried out using a cross beater mill. The sample was introduced into the grinding chamber, broken with pins, and bounced against a sieve. The sample remained in the grinding chamber until its particle size was reduced so that it passed through the sieve. Due to the high fat content of the CGB, the size of the sieve could not be selected arbitrarily; otherwise, the fat would have flowed out of the sample. For this reason, a 2.0 mm sieve was used. With the mill used, 8 kg CGB was crushed per hour. This was done at 4000 rpm.

Defatting. CO_2_ high-pressure extraction uses carbon dioxide (CO_2_) under supercritical conditions. This supercritical fluid extraction (SFE) process has the advantage over traditional solvents that the extract can be used in food or medical and pharmaceutical products subject to high product quality standards. The substance dissolved in the fluid gas is separated isobarically and isothermally. This process is used when, for example, the food product needs to be freed from extractable substances, as applied for the degreasing of green coffee beans. For this purpose, the extraction sleeve of the extraction unit was filled with 3 kg (equivalent to capacity) of ground coffee beans and extracted at a pressure of 300 bar and 50 °C. The fat content of the ground green coffee beans was then removed. The fat content of the ground green coffee beans was determined analytically in advance to calculate the expected extract quantity. The degreasing process in the extraction plant was continued (approximately 7 h) until the calculated extract quantity was approximately achieved.

### 2.5. Protease-Assisted Extraction from the Raw Materials

Protease-assisted extraction was performed to recover the protein fraction (and co-extracted polyphenols) from coffee silverskin, raw, and defatted green coffee beans. Protease-assisted extraction was performed as described in [21]. In short, the protein fractions of the coffee by-products (green beans and silver skins) were extracted by enzyme-assisted extraction using specific proteases (protease from *B. licheniformis*, trypsin, pepsin, papain, and a combination of protease from *B. licheniformis* and papain), finally obtaining peptides.

The reaction was carried out under the following conditions:

- Reaction medium: 10 mM phosphate buffer (for protease from *B. licheniformis*, papain, trypsin, and mix) or 10 mM hydrochloric acid (pepsin).
- Enzyme/substrate ratio: 1% (*w*/*w* or *v*/*w*).
- Hydraulic module: 1 to 5 (1 part of sample and 5 parts of reaction medium) for CGB, 1 to 10 for CSS.
- Hydrolysis time: 2 h.
- pH and temperature for each enzyme:
  ○ Protease from *B. licheniformis*: pH 6.5–8.5, T 60 °C.
  ○ Trypsin: pH 7–9, T 37 °C.
  ○ Pepsin: pH 2–4, T 37 °C.
  ○ Papain: pH 6–7, T 65 °C.
  ○ Mix of protease from *B. licheniformis* and papain: pH 6.5–7, T 62.5 °C.

As a control, the extraction was carried out using the same time, pH, and temperature conditions but without the enzyme.

### 2.6. SDS-PAGE of Extracts

Polyacrylamide gel electrophoresis in denaturing conditions was performed to study the protein profile (and protein integrity) in the raw materials and the obtained extracts. The solutions obtained from the aqueous, and enzyme-assisted extraction were analyzed directly. Electrophoresis was performed as described in a previous work [27] and according to the manufacturer’s instructions (Biorad, Hercules, CA, USA). Briefly, the protein samples were dissolved with 25 μL of reducing sample buffer (obtained by suitable dilution of the sample buffer XT 4× and the reducing agent XT 20× with ultrapure water). The proteins were denatured by heating to 95 °C for 5 min, then loaded onto the Criterion XT Precast gel. An electrophoretic run was performed using the XT MES run buffer, applying a potential of 150 V to the Criterion cell for 45 min.

### 2.7. Analysis of the Total and Free Amino Acid of the Extracts

Total amino acid analysis was performed to determine the nutritional value of proteins in the raw materials and extracts obtained, in terms of essential amino acids (amino acid chemical score). The free amino acid content was determined in the extracts obtained to study the intensity of proteolysis during the extraction process. Total amino acids were determined after acid hydrolysis, as previously described [28], with slight modifications (all reagent volumes increased five times, calibration curve concentrations from 6.25 to 100 µM). Briefly, the samples were hydrolyzed with 6 M HCl, added to the nor-leucine internal standard, filtered, and suitably diluted with distilled water. The samples were then derivatized with the AccQ Tag Fluor reagent kit (Waters, Milford, MA, USA) and the amino acids were quantified with RP-UPLC/ESI-MS (Waters, Milford, MA, USA) in single ion recording mode.

For the analysis of free amino acids, 10 µL of sample were derivatized with the AccQ Tag Fluor reagent kit and analyzed with RP-UPLC/ESI-MS as previously described [28].

### 2.8. Degree of Racemization of the Amino Acids of the Extracts

The degree of racemization was determined to evaluate the quality of the proteins in the raw materials and extracts obtained. A high degree of racemization decreases the nutritional value of the proteins, and is an indication of microbial activity or severe processing conditions. The degree of racemization was determined by GC-MS after acid hydrolysis and derivatization, as previously described [29]. Briefly, a suitable volume of the solution from acid hydrolysis was dried and derivatized first with 2 M HCl in 2-propanol, then dried again and derivatized with trifluoroacetic anhydride in dichloromethane. After drying and reconstitution with dichloromethane, the samples were analyzed in a GC-MS system using a Chirasyl-Val column (Agilent, Santa Clara, CA, USA).

### 2.9. Protein Identification

Protein identification was performed to identify the main proteins in the raw materials. The electrophoretic bands were gel digested with trypsin after reduction with dithio-threitol and alkylation with iodoacetamide and analyzed by liquid chromatography coupled with mass spectrometry (RP-µHPLC-LTQ-Orbitrap, Thermo Fisher Scientific Waltham, MA USA), as described in a previous work [27]. Protein identification was performed using Peaks studio software (Bioinformatics Solutions Inc., Waterloo, ON, Canada)) and using *Coffea* as the database.

### 2.10. Biological Activities of the Extracts

The possible bioactivity of the extracts was determined by studying their antioxidant and antityrosinase activity. The extracts were resuspended in MilliQ water at a 200 mg/mL final concentration before biological activity determination.

The extract’s in vitro antioxidant activity was measured using the 20-azinobis-3-ethylbenzothiazoline-6-sulfonic acid (ABTS) method with minor modifications [30]. The results are expressed as ascorbic acid equivalents per g of dry weight (mg AAeq/g DW) by means of a dose–response calibration curve (between 0 and 2 µg of amino acid (AA)).

Antityrosinase activity was assessed by an optimized tyrosinase enzyme inhibition assay [31]. The kinetics of brown color formation was evaluated by Abs measurement (490 nm) in reactions containing 10 U of tyrosinase and 2 mM L-DOPA (the substrate) in the presence of the sample. The results are expressed as kojic acid (KA, a well-known tyrosinase inhibitor) equivalents per g of DW (mg KAeq/g DW) by means of a calibration curve (between 1 and 10 mg of KA).

### 2.11. Antimicrobial Activity of the Extracts

Antimicrobial activity was determined to evaluate the potential application for inclusion in feed for monogastrics. Antimicrobial extracts are highly interesting for this application, as they are potential alternatives for conventional antibiotics, which are still commonly used in today’s livestock production practices. The antimicrobial activity of the extracts was therefore determined in a monogastric stomach simulation model. A 20% feed suspension was made, analogous to monogastric livestock animals’ in vivo stomach conditions. To this suspension, the extract was added. The pH was controlled and set to pH 3.50 with HCl 1 N as is the case in vivo for the control and the trial group. A pathogenic strain was added to the blank and test suspensions at *t* = 0 h. As pathogen, an exponential culture of *E. coli* F4 and *Streptococcus suis* (standard organisms for Gram-negative and Gram-positive pathogens, respectively) was used, at a dose of 10^6^–10^7^ CFU/g feed. The suspension was incubated under stirring for 3 h at 37 °C, to mimic the time effect in the stomach of the monogastric animal. Dilution series and bacterial counts were performed at the start (*t* = 0 h) of the experiment and after 3 h of incubation. Results are expressed as the relative kill-off related to a feed without extract.

### 2.12. Statistical Analysis

To highlight significant differences, statistical analysis was performed using IBM SPSS Statistic software (v. 26) (Chicago, IL, USA). The ANOVA single factor test was applied to analyze the centesimal analysis data, with the significance threshold set at 0.05, with the aim of comparing the composition of the two raw materials (CGB and CSS). The Kruskal–Wallis test was applied to analyze data from the analysis of phenolic compounds and caffeine, with a significance threshold set at *p* < 0.05

The aim was to compare the content of these compounds between the two raw materials (CGB and CSS), and between the two extracts (degreased CGB and CSS) obtained with enzyme-assisted extraction. Regarding the protein extraction efficiency, the homogeneity of the variance of the data was verified with the Levene test and the Kruskal–Wallis test was applied to evaluate statistically significant differences between the samples (*p* < 0.05).

## 3. Results and Discussion

### 3.1. Molecular Characterization of By-Product Composition

Two different coffee by-products were considered: defective coffee green beans (CGBs) and coffee silver skins (CSSs). All samples belonged to the *Coffea arabica* species. CGBs are discarded by roasters because they do not comply with the required quality standards. CSS, the fine integument that covers the coffee seeds, is discarded during the production of coffee, mainly during roasting. The raw composition of the coffee by-products is reported in Table 1.

The water content of the CSS was found to be higher than that of CGB. As a matter of fact, it was possible to store the CGB at room temperature, while the CSSs needed to be stabilized to avoid the formation of mold (for example, by freezing). The crude protein content was found to be comparable between the two by-products (*t*-test, *p* < 0.05). However, it must be considered that the presence of caffeine (being a nitrogenous compound) can affect this result in both by-products. In addition, in CSS, the heat-induced reactions to proteins (e.g., Maillard reaction) may have transformed the proteins into pyrazines and other non-protein nitrogenous compounds. Both factors can lead to an overestimation of the actual protein content determined with the Kjeldahl method. All mean values of proximal analysis were not significantly different (*p* < 0.05) between CGB and CSS.

**Figure 2:**
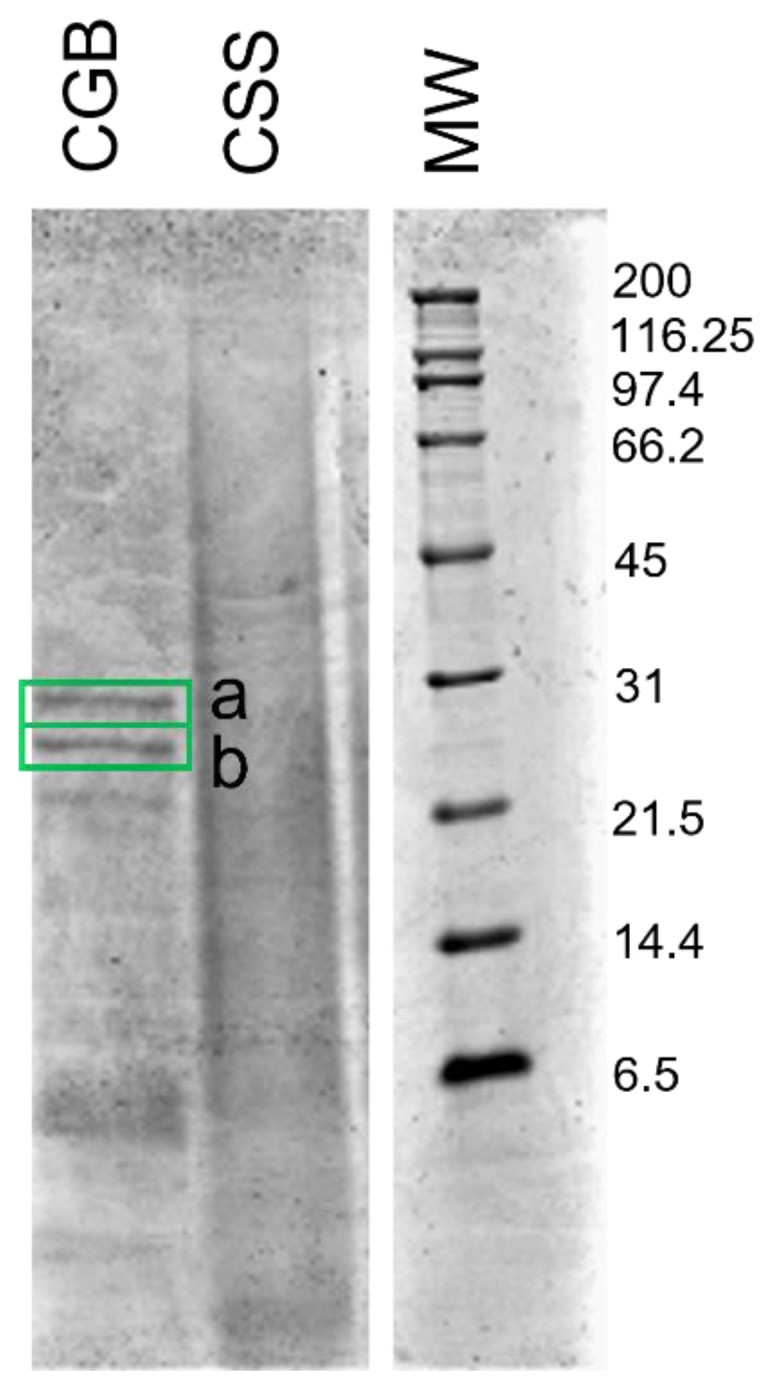
SDS-PAGE of CGB and CSS. Molecular weights of the marker are expressed in kDa. Bands identified by HRMS are highlighted.

Regarding the protein composition, the protein profile was also studied with sodium dodecyl sulphate polyacrylamide gel electrophoresis (SDS-PAGE, Figure 2). While protein bands could be identified in the CGB lane, only smears were present in CSS. The absence of defined protein bands in the latter gels is probably due to the degradation of proteins during coffee processing. In fact, the high temperature reached during roasting can induce cross-linking and degradation of proteins, resulting in the formation of high-molecular-weight insoluble compounds and compounds derived from the Maillard reaction and Strecker degradation. The two most intense bands of CGB were identified after trypsin digestion with high-resolution mass spectrometry, and were identified as 11 S storage globulins, seed storage proteins of about 55 kDa. In agreement with the literature [32], the presence of a reducing agent in the sample buffer used for the SDS-PAGE generated two separate polypeptides of approximately 33 and 24 kDa. Hence, gel electrophoresis indicates good preservation of the protein fraction of the defective CGB, which is comparable to that of the non-defective green beans. CSS, on the other hand, did not show a good protein fraction, which was not comparable to that of the original green coffee beans.

To further investigate the presence of possible protein hydrolysis, the degree of hydrolysis (DH%) of the samples was determined with OPA (*o*-pthaldialdehyde). To our knowledge, this is the first time that the degree of protein hydrolysis has been determined in these by-products. The DH% was very low for both raw materials (1.9 ± 0.1 for the CGB and between 0.4 and 0.9% for the CSS). This means that a negligible amount of free amino acids and peptides are formed by peptide bond hydrolysis. The low DH% for CGB confirms the protein integrity observed in gel electrophoresis. For CSS, the low DH% together with the absence of clearly defined protein bands on the gel may indicate the presence of protein-derived high molecular weight (MW)products (polymerization or aggregation phenomena), or extensive degradation, leading to a complex mixture of degraded nitrogen compounds.

As a further indicator of the protein quality of coffee by-products, the degree of racemization was also determined in all samples. Following a drastic heat treatment, or very acidic or alkaline pH conditions, a fraction of the amino acids, normally present in food in the l-form, changes its optical configuration and generates d-amino acids. The amount of d-amino acids formed is proportional to the harshness of the conditions. The degree of racemization is reported in Table 1, reporting the most abundant five amino acids found in the d-form. CGB, in agreement with previous results, showed a very low value of d-amino acids (less than 5%), confirming the high protein integrity also observed with SDS-PAGE and DH%. On the contrary, CSS showed higher values of d-Glu and, even more, d-Asp (close to 10%). The high temperatures used for roasting can explain this increased amount of d-Glu and d-Asp, as aggressive heat treatments can induce a certain degree of racemization. Consistently, very low racemization values were found for green coffee [33]. Going into more detail on the composition of the protein fraction, the amino acid profile is shown in Table S1.

The real protein content (determined as the sum of the individual amino acids) was found to be 8.8% (on dry matter, g DW) for CGB, and between 7.1 and 7.2% for the CSS. This is quite different from the values determined by Kjeldahl for both matrices (see Table 1 above), indicating the presence of relevant amounts of non-protein nitrogen, mainly due to caffeine, and, in CSS, also products derived from amino acid degradation (pyrazines, melanoidins, and other compounds). The values of the protein content found in the present work determined with the Kjeldahl method or with the analysis of total amino acids are in perfect agreement with those previously found in the literature in the defective CGB (11–17% on a dry basis with the Kjeldahl method, and 8–9% true protein value) [34].

The nutritional value of the proteins, calculated as an amino acid score, was found to be 0.5 for CGB (limiting amino acid Met) and between 0.4 and 0.5 for CSS (limiting amino acid Lys). Lys is sensitive amino acid, which easily degrades under roasting conditions, as it can be glycated during the Maillard reaction. The calculated amino acids scores are consistent with those of plant-based proteins.

The by-product polyphenols and caffeine contents are reported in Table 1. On average, CGB was found to be 5.9-, 3.9-, and 1.9-times richer in total polyphenols, flavonoids, and caffeine, respectively, as compared to CSS. The total phenolic content of defective CGB (27.22 ± 1.03 mg GA eq/g DW) is lower than that previously observed in non-defective CGB (40.14 ± 1.11 mg GA eq/g DW) [35], even though many factors can affect this value (genetic, environmental, storage, etc.). Tannin contents were not significantly different, and flavanols were 2.3-times more abundant in CSS.

Overall, both matrices, and particularly CGB, showed a promising composition for consideration for further valorization.

### 3.2. CGB and CSS Pretreatment

Initially, the CGB by-products underwent a deep pretreatment. First, CGB surfaces were scraped in order to improve the hygienic conditions of the sample. Then, to ensure even extraction, the beans were also ground. To prevent fat separation, the temperature during grinding was kept low, and thus the sample could not be ground to the desired fineness. Thus, to eventually improve further processability, CGBs also underwent alternative processing, including a defatting step of the grains. The details of both pretreatments are reported in the experimental section. In this way, pretreated non-defatted CGB and pretreated defatted CGB were obtained. As far as CSSs were concerned, no extensive pre-treatment, apart from grinding, was applied.

### 3.3. Protein Extraction

Enzyme-assisted aqueous extraction (EAE) was applied to extract the protein fraction from these raw materials. EAE exploits the action of proteolytic enzymes that breaks down peptide bonds, releasing peptides and amino acids that have improved solubility compared to whole proteins. Several enzymes were tested: *Bacillus licheniformis* protease, papain, pepsin, trypsin, and a mixture of *Bacillus licheniformis* protease and papain. The same extractions were performed precisely in the same conditions used for every enzyme extraction (temperature, pH, time) but without the addition of the enzymes, in order to evaluate the efficiency of simple aqueous extraction as compared to the use of enzymes.

**Figure 3:**
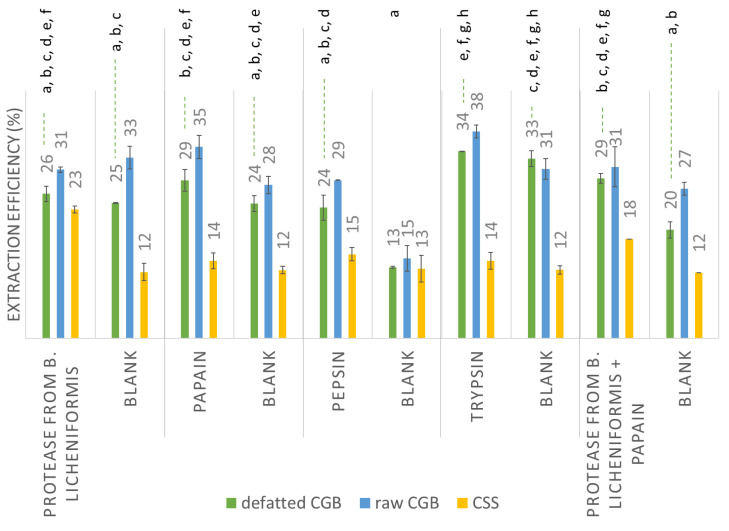
Extraction efficiency of aqueous extraction (blank) and enzyme-assisted extraction on coffee by-products. The extraction efficiency of the water extraction was measured under the same pH and temperature conditions of the respective EAE. The same letter means statistically equal values (*p* < 0.05, Kruskal–Wallis test with independent samples) among the defatted CGB extraction efficiencies. No significant differences were found for raw CGB or CSS. The pH and temperature for each extraction were: *Bacillus licheniformis* protease pH 6.5–8.5, T 60 °C; trypsin pH 7–9, T 37 °C; pepsin pH 2–4, T 37 °C; papain pH 6–7, T 65 °C; mixture of *Bacillus licheniformis* protease and papain pH 6.5–7, T 62.5 °C.

Extraction efficiencies were calculated as the amount of proteins extracted related to the initial amount of protein in the feedstock (Figure 3). While the CSSs were extracted as such after grinding, CGB were first cleaned and ground as reported above and then the extraction process was carried out both on the raw CGB (only cleaned and ground) and on the defatted CGB (cleaned, ground, and defatted).

The above data show that fat removal was not helpful, as the protein extraction efficiencies were always lower for defatted CGB. Comparing the extraction efficiency in the presence and in the absence of the proteolytic enzymes, it is evident that the two processes gave very similar results in both raw and defatted CGB. Additionally, in a previous study, the increase in protein extractability using alkaline or neutral proteases was small [19]. This might indicate a limited accessibility of proteases to proteins (given the high fiber content of CGB) or a possible interference of the high polyphenol content with the enzyme activity. For CSS, in general, there was no significant difference between the extraction efficiency with or without enzymes, with the sole exception of *Bacillus licheniformis* protease, which improved the extraction yield by 50%. Overall, both with and without enzymes, the protein extraction efficiencies appeared to be quite low, never exceeding 37%. The lower extraction yields obtained for CSS compared to CGB are consistent with the polymerization and degradation of proteins during roasting, leading to high-molecular-weight insoluble compounds.

### 3.4. Characterization of Proteins in the Extracts

**Figure 4:**
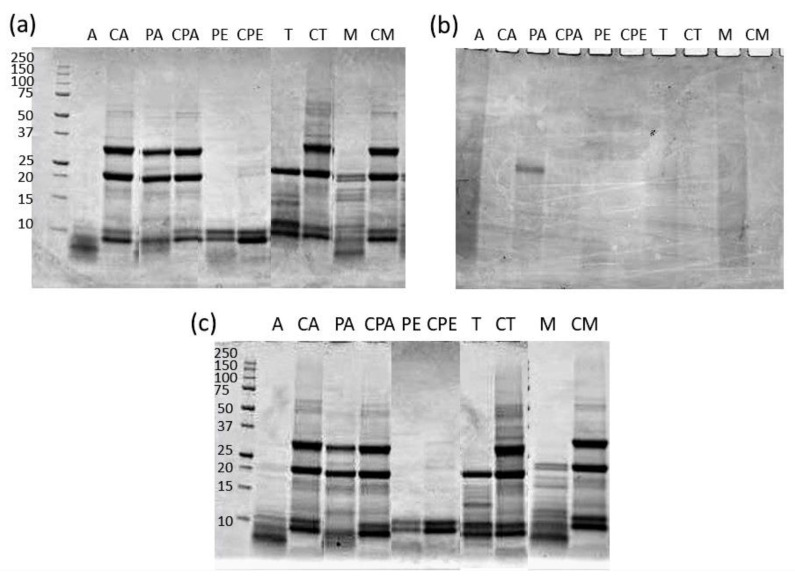
SDS-PAGE of the protein extracts obtained with enzyme-assisted extraction and aqueous extraction from: (**a**) defatted CGB, (**b**) CSS, (**c**) raw CGB. A: Bacillus licheniformis protease, PA: papain, PE: pepsin, T: trypsin, M: mix (Bacillus licheniformis protease and papain). The same codes preceded by “C” (control) indicate the aqueous extraction carried out under the same pH and temperature conditions as the respective enzyme-assisted extraction but without enzymes. The molecular weight marker is on the left (kDa).

The protein profile of the extracted fractions is shown in Figure 4.

For CGB, the protein profile was found to be the same in raw (Figure 4c) and defatted (Figure 4a) CGB, confirming that the addition of a defatting step did not affect the protein composition. The protein bands were found to be similar for all aqueous extractions performed at pH > 6 (CA, CPA, CT, and CM), with two prominent bands of about 20 and 30 kDa and some other low-molecular-weight bands (about 10 kDa).

In the case of aqueous extraction at acid pH (CPE), the two most intense bands disappeared. This could be due to the lower solubility of these proteins at the low pH (2) used for this extraction. Accordingly, the extraction performed at the same pH with pepsin (PE) also led to protein precipitation and consistently extracted less protein, producing very poor fractions with only low-molecular-weight polypeptides.

As per the other enzymes used, in the case of papain, the enzymatic extraction yielded protein profiles very similar to the extraction performed in the same conditions without the enzyme, confirming that in this case, the activity of the enzyme was very limited. Additionally, in the case of trypsin and the mix *Bacillus licheniformis* protease + papain, discrete bands could be observed after extraction, indicating a non-complete hydrolysis, and thus a low enzyme efficiency. Only when using *Bacillus licheniformis* protease did the protein bands disappear, leaving low-molecular-weight polypeptides, and thus indicating that the proteolytic activity was effective. However, as already said above, this proteolytic efficiency did not translate to an increased protein extraction in the case of CGB.

In the case of CSS (Figure 4b), all the protein extracts obtained, independently from the technique used, showed an absence of defined bands, consistent with what was observed for the initial feedstock and protein degradation already observed.

### 3.5. Protein and Amino Acid Content of the Enzymatic Extracts

Due to the comparable extraction yield and protein content of the AE extracts and respective EAEs, together with the similar protein profile on SDS-PAGE, the total amino acid profile of the obtained extracts was characterized only for the EAE extracts (Table S1). Since this is one of the first attempts to use enzymes to extract proteins from green coffee, the work focused on studying the properties of these extracts, and typically bioactivity is related more to the presence of peptides rather than whole proteins.

First, the amino acid content was used to calculate the actual amount of protein in the extracts (calculated as the sum of the total amino acids, Table 2).

The total protein content was similar among the different enzymes tested, and between non-defatted and defatted CGB. In general, the amount of proteins in the extracts was found to be higher than in the raw material. The real protein content of CGB extracts, consistent with the above results, was always found to be much higher than that of CSS extract.

As per the specific amino acid distribution, CGB extracts were, in general, pretty similar to the starting raw materials, even if some amino acids showed significant variations: the greatest fluctuations concerned Ala (14–27% higher in extracts), Ile (20–31% lower in extracts), Lys (25–46% higher in extracts), and Cys (19–53% higher in extracts). On the other hand, CSS extract was different to the raw material, with a lower amount of Pro, Val, Leu, and Phe, and higher amounts of Lys and Tyr.

The amino acid score of the EAE extracts was calculated using human milk protein as a reference and was approximately 0.5–0.6 for the CGB extracts (limiting amino acid Ile) and approximately 0.6–0.7 for the CSS extracts. While for CGB, the amino acid score was comparable for the different enzymes, for CSS, the *Bacillus licheniformis* protease provided a higher AAS (0.7, limiting amino acid Lys).

The free amino acids content was also measured to further investigate the proteolytic action of the enzymes used for EAE (Table 3, free amino acids expressed as % of total amino acids).

The amount of free amino acids in the CGB extracts obtained with enzyme extraction was found to be quite high, in the range of 12–20% of the total amino acid content, and thus the samples, judging from SDS-PAGE analysis, had very low proteolytic efficiency.

Again, the amount of free amino acids in CSS was lower, consistent with a less efficient proteolytic process of the degraded proteins. This lower efficiency is attributed to the modifications that occurred in the protein fraction during roasting, which make the proteins no longer recognizable by the proteolytic enzymes.

The degree of racemization was also determined and compared with that of the starting raw material in order to verify the possible influence of the extraction process on the quality of the proteins. The racemization degree of all the extracts was found to be completely comparable to that of the starting raw material, confirming that the extraction techniques applied had a minimal impact on the integrity of the protein fraction.

The peptides in the extracts were identified by high-resolution tandem mass spectrometry. Complete lists of the identified peptides are shown in Supplementary Materials Table S2 (CGB) and Table S3 (CSS).

As far as we know, this is one of the first studies that has performed peptide identification of coffee residues. The ADVFNPR peptide from 11 S storage globulin was identified in the CGB extract, which was also previously identified in oil palm kernel glutelin-2 hydrolysates and was shown to have significant antihypertensive effects in spontaneously hypertensive rats [36] and antioxidant activity [37]. From the analyses of the peptides, it was observed that in the CSS extract (and not in the CGB), half of the peptides containing methionine have methionine in its oxidized form (+16 Da), also indicating a harsh treatment of the CSS (presumably during roasting), which further decreases the nutritional value of the proteins.

The characterization of EAE-extracted proteins from defective CGB and CSS reported here brings new knowledge in this field, since most of the work on these coffee by-products has focused on other components (bioactives, fibers, etc.), while very little information is available on amino acids, peptides, and proteins.

### 3.6. Polyphenol Content and Biofunctional Properties of the Enzymatic Extracts

The following analyses of the biofunctional properties of CGB and CSS EAE extracts were carried out on the samples obtained with *Bacillus licheniformis* protease from extracted defatted CGB. The choice of this sample containing this specific enzyme as an extracting adjuvant was due to the consideration that usually, bioactive properties are due to low-molecular-weight peptides and not to whole proteins. From the gel electrophoresis (Figure 4), the enzyme in which fewer protein bands were present, and thus the sample for which more peptides are expected to be present, was the one obtained with *Bacillus licheniformis* protease.

Total polyphenol analysis was also performed (Table 4), and as expected, in addition to peptides, CGB and CSS extracts contained 4 and 3 times more total polyphenols than the respective initial by-product (Table 1).

The antioxidant and antityrosinase activities were measured on these two samples. The two selected extracts exerted useful biofunctional properties (Table 4), due to the synergistic action of peptides and polyphenols. As expected, the in vitro antioxidant activity was found to be 4 times higher in the CGB sample than in the CSS, due to the higher polyphenol content, while the antityrosinase property is somewhat better in the CSS extract. The total polyphenol content is in line with that of other aqueous extracts obtained from CGB (169.00 ± 3.06 mg GA eq/g DW) [38] or hydroalcoholic extract from CGB (8.82  ±  0.12% *w*/*w*, gallic acid) [11]. Some discrepancies may be due, in addition to the extraction method, to the variety of coffee, the place of harvest, the storage conditions, and, of course, the fact that we compared defective CGB with commercial ones. Considering the percentage of total phenolic compound equivalents to gallic acid in hydroalcoholic extracts from green coffee oil extraction residues (11.5–14.7% in dry extracts) [39], the values found here for defective CGBs are slightly lower but are still in line. On the contrary, the total polyphenol content of the CSS extract was found to be much lower in the EAE extract than in hydroalcoholic extracts studied in the literature (10.75  ±  0.58% *w*/*w*, gallic acid) [11]. Even considering aqueous extractions, the value of the total phenol content found in the present work is lower than other previously determined values in the literature (20.49 ± 0.27 mgGA/g of extract) [40]. However, contrary to what was previously observed in the aqueous extracts of CSS, where no inhibition of tyrosinase was observed, in this case, a low but detectable inhibition was observed, hinting at a possible role of peptides. Indeed, a possible explanation could be the presence of the *B. licheniformis* protease, which may have generated peptides with antityrosinase activity. Anyway, none of the identified peptides are yet known for any potential bioactivity (Tables S2 and S3 Supplementary Materials). There is growing interest in the recovery of nutraceutical compounds from coffee by-products, as they have shown good antioxidant and enzyme inhibitory properties: hydroalcoholic extracts of spent coffee grounds have a high antioxidant activity, and hydroalcoholic extracts of spent coffee grounds and coffee silver skins show inhibitory activity towards a series of enzymes (cholinesterases, amylase, glucosidase, and tyrosinase). Among coffee industry by-products with antioxidant activity, silver skin was found to have the highest phenolics, followed by spent waste and cherry husk after pre-treatment with viscozyme [41]. Hydrothermal extracts of spent coffee grounds showed good antioxidant and tyrosinase-inhibiting capacity, thus suggesting a possible reuse in cosmetic products. Therefore, the results presented here contribute to an investigation of the interesting properties of coffee by-products as a source of bioactive extracts and ingredients, from a circular economy perspective.

### 3.7. Antimicrobial Activity of the Extracts

The antimicrobial activity of the extracts was also determined in a monogastric stomach simulation model. The antimicrobial activity against two pathogens, *E. coli* F4 and *Streptococcus suis*, was determined and expressed as the relative kill-off related to a feed without extract. The results of the antimicrobial activity test at two dosages (an equivalent dosage of 1.25 and 12.5 kg/MT) are presented in Table 5.

The defatted CGB extract showed promising antimicrobial activity against both the Gram-negative and Gram-positive pathogens. An increase in the inclusion level only gave rise to a marginal improvement in activity. This contrasts with the CSS extract, where a 10 times higher inclusion level caused an important increase in antimicrobial activity. The CSS extract showed promising antimicrobial activity against *Streptococcus suis*, the Gram-positive pathogen, at the highest inclusion level. Nanoemulsions containing oil and aqueous extract of green coffee beans have previously shown antibacterial activity against *S. aureus*, *S. epidermidis*, and *E. coli* [42], and subcritical water hydrolysates of green coffee beans have generally shown good antimicrobial activity against Gram-positive bacteria (especially *S. aureus*) [43]. Thus, the results obtained confirm the promising antibacterial properties of CGB extracts. The lower antibacterial activity of CSS is also partially confirmed in the literature. In fact, in previous works, silverskin extracts did not inhibit the growth of any bacterial species among those studied (*S. aureus*, *P. aeruginosa*, *E. coli*) [44]. However, even if less active than CGB, CSS showed detectable antimicrobial activity, especially at the concentration of 12.5 kg/MT. This is consistent with a previous study in which aqueous extracts of CSS had MICs in the range of 31.3–250 µg/mL against *S. aureus*, *S. epidermidis*, *E. coli*, and *K. pneumoniae*, thus indicating a potential antimicrobial effect [45]. Therefore, the results obtained, especially for CGB extracts, indicate good antimicrobial activity (kill off >50%) at a concentration (1.25 kg/MT) that is consistent with that of other antimicrobial additives commonly added to feeds.

## 4. Conclusions

In the present work, a complete characterization of two by-products of the coffee industry, i.e., defective coffee green beans (CGBs) and coffee silver skins (CSSs), was carried out. The quantity and quality of the protein fraction, and the contents of polyphenols, flavanols, flavonoids, tannins, and caffeine were assessed. Except for the tannins, CGBs showed a greater amount of all these compounds than CSSs. Furthermore, the protein fraction of the CGB is very well conserved, while a strong degradation was observed for the CSS. Consistently, enzyme-assisted and conventional protein extraction from the two matrices yielded better results for CGB rather than CSS. The enzyme-assisted extraction of CGB (using the protease from *B. licheniformis*) produced extracts with a good protein content (18%, with well-preserved proteins and good nutritional value) and also with a consistent amount of polyphenols. The CGB extract showed antioxidant, antityrosinase, and antimicrobial activities, due to their polyphenol and peptide contents. This is a promising strategy to recover defective coffee green beans by producing extracts with interesting activities for the cosmetic or feed industry. CSS extracts are less promising than CGBs, as the quantity and quality of proteins are lower; the antioxidant and antimicrobial activity is lower than that in CGB extracts, while the antityrosinase activity is comparable to that of CGB. Therefore, a valorization path oriented more towards producing bio-based composites would be more suitable for CSS. More studies are needed to correlate the observed biological properties with the actual polyphenol and peptide composition.

## Figures and Tables

**Table 1:** Raw composition of coffee by-products: proteins, fibers, sugars, and lipids are expressed as dry matter (g DW). The degree of racemization of the proteins of coffee by-products is expressed as % of d-amino acid on the sum (d + l)-amino acid. Results of proximal analysis, phenolic compounds, and caffeine contents of coffee by-products are reported as the mean ± sd and expressed on dry matter (g DW).

Component Analysis	CGB	CSS
Dry residue (%)	96.7 ± 0.0 ^a^	82.5 ± 1.6 ^a^
Proteins (% g/100 g DW)	15.3 ± 0.1 ^a^	16.3 ± 0.2 ^a^
Fibers (% g/100 g DW)	56.4 ± 0.1	69.8 ± 0.2
Sugars (% g/100 g DW)	8.0 ± 0.8 ^a^	0.4 ± 0.2 ^a^
Lipids (% g/100 g DW)	13.6 ± 0.2 ^a^	6.3 ± 0.1 ^a^
d-Ala	2.0 ± 0.3	2.8 ± 0.4
d-Asp	4.7 ± 0.4	9.0 ± 0.7
d-Glu	1.9 ± 1.2	5.5 ± 3.5
d-Phe	1.7 ± 0.3	2.9 ± 0.6
d-Lys	2.3 ± 0.9	1.7 ± 0.6
Total polyphenols (mg GA eq/g DW)	27.22 ± 1.03 ^a^	5.36 ± 0.33 ^b^
Total flavonoids (mg CAT eq/g DW)	16.29 ± 0.65 ^a^	4.81 ± 0.66 ^b^
Total flavanols (mg CAT eq/g DW)	5.26 ± 0.49 ^a^	0.90 ± 0.10 ^b^
Tannins (mg/g DW)	2.46 ± 0.40 ^a^	2.48 ± 0.14 ^b^
Caffeine (mg/g DW)	15.18 ± 0.49 ^a^	9.08 ± 0.97 ^b^

Equal letters in the same row = statistically equal data between CGB and CSS (*p* < 0.05, ANOVA single factor test). Different letters (a and b) on the same row means statistically different values.

**Table 2:** The real protein content of the extracts obtained with UAE and EAE, expressed as % on the dry weight (g DW).

Extraction Condition	Non-Defatted CGB	Defatted CGB	CSS
EAE—*Bacillus licheniformis* protease	17	16	10
EAE—papain	17	19	5
EAE—pepsin	15	15	4
EAE—trypsin	17	18	5

**Table 3:** Amount of free amino acids (% of total amino acids) in the dry protein extracts.

Extraction Condition	Raw CGB	Defatted CGB	CSS
EAE (*Bacillus licheniformis* protease)	18	18	3
EAE (papain)	17	16	11
EAE (pepsin)	21	20	6
EAE (trypsin)	15	12	4
EAE (*Bacillus licheniformis* protease + papain)	18	18	4

**Table 4:** Antioxidant, antityrosinase, and antimicrobial activities of CGB Bacillus licheniformis protease extract (equal letters = statistically equal data, *p* < 0.05, Kruskal–Wallis test). Different letters (a and b) in the same column mean statistically different values.

Coffee By-Product	Total Polyphenols (mg GA eq/g DW)	Antioxidant Activity (mg AA eq/g DW)	Antityrosinase Activity (mg KA eq/g DW)
CGB—EAE-(*Bacillus licheniformis* protease)	99.02 ± 10.13 ^a^	78.74 ± 4.90 ^a^	2.25 ± 0.16 ^a^
CSS—EAE-(*Bacillus licheniformis* protease)	12.63 ± 0.13 ^b^	18.95 ± 0.31 ^b^	2.85 ± 0.31 ^b^

**Table 5:** Analyzed antimicrobial activity of extracts.

Sample	Kill off *E. coli* F4 (%)		Kill off *S. suis* (%)	
	1.25 kg/MT	12.5 kg/MT	1.25 kg/MT	12.5 kg/MT
Defatted CGB—EAE (alcalase)	51	65	55.5	64.2
CSS—EAE (alcalase)	4.7	58.8	2.8	22.2

## Data Availability

The data are shown in the tables and figures, including the supplementary tables. Any questions regarding specific data can be directed to the corresponding author.

## Supplementary Materials

The following are available online at https://www.mdpi.com/article/10.3390/biom11111571/s1, Table S1: total amino acids composition of raw materials (% on fresh weight) and freeze-dried protein extracts. n.d.: not determined; Table S2: peptide composition of the dGCB extracted with protease from *B. licheniformis* s; Table S3: peptide composition of the CSS extracted with protease from *B. licheniformis*.
