# Supplementary material for: Extraction and Chemical Characterization of Functional Phenols and Proteins from Coffee (Coffea arabica) By-Products
Source: Biomolecules. 2021 Oct 22;11(11):1571. doi: 10.3390/biom11111571 (PMC8615506; doi:10.3390/biom11111571)
Supplement: Supplementary file 1 [file biomolecules-11-01571-s001.zip › biomolecules-1405502-supp-for final.pdf]

Table S1 Total amino acids determinationin in the enzymatic extracts

	Amino acids (% w/w)																
**Sample type**	**Gly**	**Ala**	**Ser**	**Pro**	**Val**	**Thr**	**Ile**	**Leu**	**Asp**	**Lys**	**Glu**	**His**	**Phe**	**Arg**	**Tyr**	**Cys**	**Met**
Coffee green beans	0.66 ± 0.05	0.44 ± 0.02	0.51 ± 0.01	0.53 ± 0.02	0.58 ± 0.02	0.36 ± 0.01	0.40 ± 0.02	0.90 ± 0.04	1.03 ± 0.05	0.57 ± 0.01	2.16 ± 0.09	0.06 ± 0.00	0.53 ± 0.02	0.55 ± 0.03	0.22 ± 0.01	0.21 ± 0.02	0.16 ± 0.01
Coffee silverskins	0.44 ± 0.03	0.43 ± 0.01	0.45 ± 0.01	0.46 ± 0.02	0.47 ± 0.01	0.37 ± 0.01	0.40 ± 0.01	0.65 ± 0.02	0.83 ± 0.03	0.26 ± 0.01	0.78 ± 0.01	0.06 ± 0.00	0.44 ± 0.01	0.26 ± 0.01	0.18 ± 0.02	0.19 ± 0.02	0.14 ± 0.00
CGB raw alcalase	1.21 ± 0.11	0.96 ± 0.20	1.00 ± 0.09	1.19 ± 0.31	1.08 ± 0.10	0.67 ± 0.05	0.55 ± 0.27	1.59 ± 0.11	2.04 ± 0.58	1.36 ± 0.55	4.42 ± 0.88	n. d.	0.81 ± 0.45	n. d.	0.56 ± n.d.	0.46 ± n. a.	0.25 ± n. a.
CGB raw papain	1.39 ± 0.18	0.94 ± 0.11	0.96 ± 0.02	1.11 ± 0.15	1.08 ± 0.01	0.67 ± 0.09	0.56 ± 0.31	1.58 ± 0.06	2.11 ± 0.38	1.42 ± 0.15	4.57 ± 0.35	n. d.	0.88 ± 0.23	n. d.	0.75 ± n.d.	0.61 ± n. a.	0.24 ± n. a.
CGB raw pepsin	1.17 ±0.26	0.89 ± 0.15	0.87 ± 0.12	1.08 ± 0.24	0.88 ± 0.07	0.60 ± 0.03	0.45 ± 0.23	1.28 ± 0.13	1.93 ± 0.35	1.17 ± 0.19	3.85 ± 0.36	n. d.	0.69 ± 0.18	n. d.	0.57 ± n.d.	0.47 ± n. a.	0.17 ± n. a.
CGB raw trypsin	1.23 ± 0.10	0.97 ± 0.10	0.94 ± 0.06	1.09 ± 0.10	1.06 ± 0.05	0.67 ± 0.12	0.57 ± 0.35	1.56 ± 0.06	2.20 ± 0.29	1.50 ± 0.46	4.60 ± 0.26	n. d.	0.84 ± 0.49	n. d.	0.59 ± n.d.	0.54 ± n. a.	0.25 ± n. a.
CGB raw mix (alcalase + papain)	1.15 ± 0.14	0.96 ± 0.12	0.85 ± 0.05	1.02 ± 0.11	1.00 ± 0.00	0.60 ± 0.09	0.51 ± 0.31	1.46 ± 0.01	2.27 ± 0.34	1.48 ± 0.37	4.69 ± 0.34	n. d.	0.66 ± 0.38	n. d.	0.53 ± n.d.	0.52 ± n. a.	0.22 ± n. a.
CGB defatted alcalase	1.22 ± 0.24	0.94 ± 0.17	0.90 ± 0.04	1.11 ± 0.13	1.04 ± 0.02	0.64 ± 0.09	0.55 ± 0.32	1.56 ± 0.03	2.13 ± 0.57	1.41 ± 0.44	4.32 ± 0.58	n. d.	0.82 ± 0.33	n. d.	0.69 ± n.d.	0.54 ± n. a.	0.25 ± n. a.
CGB defatted papain	1.50 ± 0.63	1.14 ± 0.46	1.58 ± 1.12	1.13 ± 0.29	1.15 ± 0.31	0.86 ± 0.31	0.57 ± 0.18	1.65 ± 0.40	2.24 ± 0.60	1.48 ± 0.41	4.65 ± 0.43	n. d.	0.92 ± 0.07	n. d.	1.20 ± n.d.	0.69 ± n. a.	0.25 ± n. a.
CGB defatted pepsin	1.33 ± 0.17	0.91 ± 0.04	0.91 ± 0.01	1.13 ± 0.12	0.91 ± 0.06	0.63 ± 0.11	0.49 ± 0.31	1.32 ± 0.01	2.00 ± 0.17	1.23 ± 0.17	4.03 ± 0.09	n. d.	0.72 ± 0.32	n. d.	0.61 ± n.d.	0.55 ± n. a.	0.18 ± n. a.
CGB defatted trypsin	1.29 ± 0.24	1.01 ± 0.03	1.02 ± 0.09	1.22 ± 0.20	1.16 ± 0.04	0.72 ± 0.05	0.62 ± 0.33	1.71 ± 0.03	2.25 ± 0.17	1.46 ± 0.11	4.71 ± 0.02	n. d.	0.97 ± 0.15	n. d.	0.76 ± n.d.	0.62 ± n. a.	0.30 ± n. a.
CGB defatted mix (alcalase + papain)	1.27 ± 0.17	0.91 ± 0.06	0.96 ± 0.10	1.12 ± 0.18	1.05 ± 0.00	0.67 ± 0.07	0.55 ± 0.31	1.52 ± 0.03	2.02 ± 0.18	1.28 ± 0.15	4.46 ± 0.01	n. d.	0.91 ± 0.28	n. d.	0.75 ± n.d.	0.53 ± n. a.	0.24 ± n. a.
CSS alcalase	0.70 ± 0.09	0.75 ± 0.01	0.72 ± 0.01	0.67 ± 0.02	0.74 ± 0.01	0.67 ± 0.02	0.71 ± 0.00	0.99 ± 0.02	1.57 ± 0.04	0.55 ± 0.01	1.47 ± 0.01	0.35 ± 0.04	0.68 ± 0.03	0.44 ± 0.02	0.53 ± 0.02	0.27 ± 0.02	0.28 ± 0.03
CSS papain	0.50 ± 0.01	0.26 ± 0.00	0.27 ± 0.01	0.23 ± 0.02	0.22 ± 0.02	0.23 ± 0.00	0.24 ± 0.00	0.27 ± 0.00	0.73 ± 0.01	0.51 ± 0.01	0.77 ± 0.03	0.26 ± 0.02	0.18 ± 0.00	0.25 ± 0.01	0.18 ± 0.00	0.30 ± n. a.	0.10 ± n. a.
CSS pepsin	0.42 ± 0.01	0.27 ± 0.00	0.28 ± 0.03	0.28 ± 0.02	0.21 ± 0.00	0.23 ± 0.01	0.22 ± 0.01	0.26 ± 0.00	0.68 ± 0.01	0.5 ± 0.01	0.63 ± 0.02	0.23 ± 0.01	0.16 ± 0.00	0.26 ± 0.02	0.17 ± 0.00	0.08 ± n. a.	0.06 ± n. a.
CSS trypsin	0.34 ± 0.03	0.28 ± 0.00	0.29 ± 0.01	0.29 ± 0.00	0.26 ± 0.02	0.25 ± 0.02	0.27 ± 0.01	0.32 ± 0.01	0.75 ± 0.01	0.47 ± 0.02	0.65 ± 0.01	0.31 ± 0.04	0.21 ± 0.03	0.35 ± 0.04	0.21 ± 0.00	0.17 ± 0.04	0.14 ± 0.03
CSS mix (alcalase + papain)	0.72 ± 0.00	0.64 ± 0.01	0.6 ± 0.02	0.58 ± 0.01	0.62 ± 0.01	0.55 ± 0.02	0.6 ± 0.02	0.79 ± 0.02	1.36 ± 0.02	0.55 ± 0.02	1.3 ± 0.04	0.32 ± 0.04	0.57 ± 0.01	0.38 ± 0.02	0.43 ± 0.00	n. a.	n. a.

n. d.: not detected n. a.: not available

Table S2 Peptide identification in the CGB enzymatic extracts

Peptide	-10lgP	Mass	Length	ppm	m/z	RT	Area	Accession	PTM
NAQEPSFRFPS	71,27	1278,5992	11	1,4	640,3078	19,24	2,15E+06	tr|O82437|O82437_COFAR:tr|P93079|P93079_COFAR:tr|Q9ZNY2|Q9ZNY2_COFAR	
NAQEPSFRFPSE	66,63	1407,6418	12	2,5	704,83	19,42	8,56E+05	tr|O82437|O82437_COFAR:tr|P93079|P93079_COFAR:tr|Q9ZNY2|Q9ZNY2_COFAR	
AQEPSFRFPSE	65,28	1293,5989	11	1,5	647,8077	19,51	3,36E+05	tr|O82437|O82437_COFAR:tr|P93079|P93079_COFAR:tr|Q9ZNY2|Q9ZNY2_COFAR	
NAQEPSFRFPSEAGLT	62,77	1749,8322	16	3,3	875,9263	23,23	4,38E+04	tr|O82437|O82437_COFAR:tr|P93079|P93079_COFAR:tr|Q9ZNY2|Q9ZNY2_COFAR	
AQEPSFRFPS	60,23	1164,5563	10	1,7	583,2864	19,24	7,08E+05	tr|O82437|O82437_COFAR:tr|P93079|P93079_COFAR:tr|Q9ZNY2|Q9ZNY2_COFAR	
NAQ(+.98)EPSFRFPS	56,49	1279,5833	11	0,4	640,7991	22,94	5,09E+04	tr|O82437|O82437_COFAR:tr|P93079|P93079_COFAR:tr|Q9ZNY2|Q9ZNY2_COFAR	Deamidation (NQ)
DSIDRGNDAWKGM	54,23	1463,6462	13	1,7	732,8316	14,11	2,54E+05	tr|A0A068TKQ7|A0A068TKQ7_COFCA:tr|A8Y5V3|A8Y5V3_COFAR	
N(+.98)AQEPSFRFPS	48,79	1279,5833	11	0,4	640,7991	22,94	5,09E+04	tr|O82437|O82437_COFAR:tr|P93079|P93079_COFAR:tr|Q9ZNY2|Q9ZNY2_COFAR	Deamidation (NQ)
ADVFNPR	44,92	817,4082	7	2,1	409,7122	9,58	7,59E+04	tr|O82437|O82437_COFAR:tr|P93079|P93079_COFAR:tr|Q9ZNY2|Q9ZNY2_COFAR	
GFPDAVNHPNFPS	42,28	1397,6364	13	2,7	699,8273	19,84	6,83E+04	tr|A0A068VDG8|A0A068VDG8_COFCA	
QEPSFRFPS	38,54	1093,5192	9	1,1	547,7675	19,14	5,90E+05	tr|O82437|O82437_COFAR:tr|P93079|P93079_COFAR:tr|Q9ZNY2|Q9ZNY2_COFAR	
VDKGTVELPGTN	38,16	1228,6299	12	-1,5	615,3213	13,26	3,53E+04	tr|A0A068V3J7|A0A068V3J7_COFCA:tr|A0A068UAS9|A0A068UAS9_COFCA	
QEPSFRFPSE	36,87	1222,5618	10	1,2	612,2889	19,41	2,39E+05	tr|O82437|O82437_COFAR:tr|P93079|P93079_COFAR:tr|Q9ZNY2|Q9ZNY2_COFAR	
SENIGLPQE	35,42	985,4716	9	1,5	493,7438	17,3	2,03E+05	tr|O82437|O82437_COFAR:tr|P93079|P93079_COFAR:tr|Q9ZNY2|Q9ZNY2_COFAR	
SIDRGNDAWKGM	35,32	1348,6194	12	2,2	675,3185	11,59	2,52E+04	tr|A0A068TKQ7|A0A068TKQ7_COFCA:tr|A8Y5V3|A8Y5V3_COFAR	
NAQEPSFRFP	33,55	1191,5673	10	1,2	596,7916	20,95	2,05E+04	tr|O82437|O82437_COFAR:tr|P93079|P93079_COFAR:tr|Q9ZNY2|Q9ZNY2_COFAR	
KTNDNAMINPL	31,18	1229,6074	11	1,1	615,8116	17,16	8,82E+04	tr|O82437|O82437_COFAR:tr|P93079|P93079_COFAR:tr|Q9ZNY2|Q9ZNY2_COFAR	
NAQEPSFRFPSEA	30,18	1478,679	13	7	740,3519	19,72	2,21E+04	tr|O82437|O82437_COFAR:tr|P93079|P93079_COFAR:tr|Q9ZNY2|Q9ZNY2_COFAR	
YIGTPGKGIL	29,57	1017,5858	10	2,3	509,8013	18,13	1,91E+04	tr|A0A068V3J7|A0A068V3J7_COFCA:tr|A0A068UAS9|A0A068UAS9_COFCA	
DSIDRGNDAW	29,07	1147,4894	10	2,1	574,7532	13,67	1,09E+05	tr|A0A068TKQ7|A0A068TKQ7_COFCA:tr|A8Y5V3|A8Y5V3_COFAR	
DVGRPGVQDPGVG	28,31	1251,6207	13	2,4	626,8192	11,05	1,46E+04		
LVGKDPTDQTGID	28,13	1357,6725	13	2,3	679,8451	11,33	8,07E+03		
SDKVQIF	27,35	835,444	7	0,8	418,7296	16,36	2,04E+04	tr|A0A068VDG8|A0A068VDG8_COFCA	
SE(+55.92)NIGLPQ	26,62	912,3487	8	1	457,1821	16,68	2,68E+05	tr|O82437|O82437_COFAR:tr|P93079|P93079_COFAR:tr|Q9ZNY2|Q9ZNY2_COFAR	
SEEAEELKYGRQEALLLSEQSQQ	26,07	2664,2878	23	1,3	889,1044	20,92	2,90E+04	tr|O82437|O82437_COFAR:tr|P93079|P93079_COFAR	
RQPGPIPGIN	23,88	1047,5825	10	2	524,7996	14,64	9,51E+03		
SAYGAGVP	22,79	720,3442	8	-9,2	361,1761	41,76	2,47E+04		
QLSAERGFLY	22,66	1182,6033	10	5,2	592,312	19,98	1,00E+04	tr|O82437|O82437_COFAR:tr|P93079|P93079_COFAR:tr|Q9ZNY2|Q9ZNY2_COFAR	
FVVCEPGT	19,66	850,3895	8	0,2	426,2021	35,94	1,02E+05		
QENIGRVL	19,59	927,5137	8	1,3	464,7647	13,68	2,86E+04	tr|A0A068TKQ7|A0A068TKQ7_COFCA:tr|A8Y5V3|A8Y5V3_COFAR	

LASSLNYIGGT	19,32	1094,5608	11	-6,3	365,8586	11,1	1,60E+05		
NLPDGGCGNK	18,38	973,4287	10	-5,8	487,7188	31,55	1,17E+05		
QSPTIPQ	18,13	769,397	7	0,5	385,706	16,97	2,68E+04		
HPPLCIVSDIF	17,93	1239,6322	11	4,1	620,8259	18,59	1,26E+05		
AVNHPNFPS	17,5	981,4668	9	1,6	491,7415	9,88	1,57E+04	tr|A0A068VDG8|A0A068VDG8_COFCA	
KDEGEPPK	17,3	898,4396	8	0,9	450,2275	17,51	2,20E+04		
KCSGEPKGQ	17,03	932,4385	9	6,8	467,2297	18,57	1,00E+05		
CLDCFSAPEEVHD	17,03	1463,5697	13	-3,9	732,7893	19,42	8,54E+04		
ESGDNHEQ	16,48	914,3366	8	7,4	458,179	16,64	9,71E+04		
ENPQNSKPDDDAS	15,82	1415,5801	13	-0,3	708,7971	12,11	1,57E+04		
NDGD(+55.92)VPL	15,79	784,2538	7	1,6	393,1348	15,73	2,63E+05	tr|O82437|O82437_COFAR:tr|P93079|P93079_COFAR:tr|Q9ZNY2|Q9ZNY2_COFAR	
ALGGFGVGLGAGV	15,45	1073,5869	13	1	537,8013	18,81	2,18E+04		
SGYGGSKS(+79.97)G	15,37	878,3171	9	-8,6	440,162	26,05	6,54E+04	tr|A0A068TKQ7|A0A068TKQ7_COFCA	Phosphorylation (STY)
SE(+55.92)NIGLPQE	15,1	1041,3914	9	1,3	521,7036	17,3	1,91E+05	tr|O82437|O82437_COFAR:tr|P93079|P93079_COFAR:tr|Q9ZNY2|Q9ZNY2_COFAR	
LSDHNGSS	15,02	815,3409	8	-4,7	408,6758	27,97	1,30E+05		

Table S3 Peptide identification in the CSS enzymatic extracts

Peptide	-10lgP	Mass	Length	ppm	m/z	RT	Area Sample	Accession	PTM
IIDSGITPGHPSFSDE	106,76	1670,7787	16	-0,8	836,3959	18,33	6,17E+06	tr|A0A068UNL8|A0A068UNL8_COFCA	
GIIDSGITPGHPSFSDE	98,59	1727,8002	17	-0,3	864,9071	21,33	1,60E+06	tr|A0A068UNL8|A0A068UNL8_COFCA	
VIDTGITPDHPSFSDE	92,13	1728,7842	16	1,1	865,4003	18,39	2,34E+06	tr|A0A068UNA6|A0A068UNA6_COFCA	
GHVNPPRAIDPGLT	91,04	1442,7629	14	-0,8	722,3882	13,81	1,19E+06	tr|A0A068UNL8|A0A068UNL8_COFCA	
GHVNPPRAIDPGLTY	88,35	1605,8263	15	-2	803,9188	16,84	1,11E+07	tr|A0A068UNL8|A0A068UNL8_COFCA	
GAGHVNPPRAIDPGLTY	84,36	1733,8849	17	-0,4	867,9494	17,1	2,12E+06	tr|A0A068UNL8|A0A068UNL8_COFCA	
DSGITPGHPSFSDE	81,71	1444,6106	14	0,1	723,3126	15,81	1,51E+06	tr|A0A068UNL8|A0A068UNL8_COFCA	
GVIDTGITPDHPSFSDE	80,65	1785,8057	17	1,8	893,9117	20,34	1,29E+05	tr|A0A068UNA6|A0A068UNA6_COFCA	
FIVGPIPINDGLGKE	76,28	1567,861	15	0,1	784,9379	27,64	3,98E+05	tr|A0A068V9U7|A0A068V9U7_COFCA:tr|A0A068TQU2|A0A068TQU2_COFCA	
SIQPVAPADPHVV	75,7	1328,7089	13	-0,6	665,3613	17,02	3,41E+06	tr|A0A3P8MYP6|A0A3P8MYP6_COFCA:tr|A0A068VCQ0|A0A068VCQ0_COFCA	
IIDSGITPGHPSFS	75,1	1426,7092	14	-0,4	714,3616	18,18	1,56E+06	tr|A0A068UNL8|A0A068UNL8_COFCA	
VNPPRAIDPGLTY	72,09	1411,746	13	-0,4	706,88	19,75	6,34E+06	tr|A0A068UNL8|A0A068UNL8_COFCA	
DLLGPKPGQPLDE	71,58	1377,714	13	-0,4	689,864	19,58	6,78E+06	tr|A0A068UNL8|A0A068UNL8_COFCA	
IIDSGITPGHPS	71,31	1192,6088	12	-0,4	597,3114	12,05	1,14E+07	tr|A0A068UNL8|A0A068UNL8_COFCA	
GITPGHPSFSDE	71,26	1242,5516	12	-1,4	622,2822	13,45	8,43E+06	tr|A0A068UNL8|A0A068UNL8_COFCA	
SIQPVAPADPH	69,47	1130,572	11	0,5	566,2936	10,31	1,62E+06	tr|A0A3P8MYP6|A0A3P8MYP6_COFCA:tr|A0A068VCQ0|A0A068VCQ0_COFCA	
GGPSKLPFFDDPIAL	67,89	1572,8187	15	-1,3	787,4156	33,44	6,78E+06	tr|A0A068UNL8|A0A068UNL8_COFCA	
GIIDSGITPGHPSFS	67,69	1483,7307	15	-0,9	742,8719	21,33	4,46E+05	tr|A0A068UNL8|A0A068UNL8_COFCA	
IDSGITPGHPS	64,71	1079,5247	11	0,1	540,7697	7,76	6,74E+04	tr|A0A068UNL8|A0A068UNL8_COFCA	

<table>
<tr><td>GAGHVNPPRAIDPGLT</td><td>64,4</td><td>1570,8215</td><td>16</td><td>-0,6</td><td>786,4176</td><td>14,24</td><td>4,78E+05</td><td>tr|A0A068UNL8|A0A068UNL8_COFCA</td><td></td></tr>
<tr><td>VGDPRQPNGEDM(+15.99)GNFY</td><td>64,17</td><td>1810,7581</td><td>16</td><td>0</td><td>906,3863</td><td>13,79</td><td>7,08E+05</td><td>tr|A0A068VNV4|A0A068VNV4_COFCA</td><td>Oxidation (M)</td></tr>
<tr><td>RLPGQPDGVDFN</td><td>61,62</td><td>1313,6364</td><td>12</td><td>-1,4</td><td>657,8245</td><td>17</td><td>4,41E+05</td><td>tr|A0A068TZK8|A0A068TZK8_COFCA:tr|A0A068TYK8|A0A068TYK8_COFCA</td><td></td></tr>
<tr><td>SGITPGHPSFSDE</td><td>60,14</td><td>1329,5836</td><td>13</td><td>-1</td><td>665,7984</td><td>13,63</td><td>4,52E+06</td><td>tr|A0A068UNL8|A0A068UNL8_COFCA</td><td></td></tr>
<tr><td>WVGDPRQPNGEDM(+15.99)</td><td>59,3</td><td>1515,6412</td><td>13</td><td>-1,9</td><td>758,8264</td><td>11,18</td><td>8,33E+05</td><td>tr|A0A068VNV4|A0A068VNV4_COFCA</td><td>Oxidation (M)</td></tr>
<tr><td>GKLIGDPSFGLPF</td><td>56,72</td><td>1346,7234</td><td>13</td><td>-0,1</td><td>674,3689</td><td>31,37</td><td>2,42E+06</td><td>tr|A0A068VNV4|A0A068VNV4_COFCA</td><td></td></tr>
<tr><td>WVGDPRQPNGEDM(+15.99)GNFY</td><td>55,95</td><td>1996,8373</td><td>17</td><td>-1,7</td><td>999,4242</td><td>19,28</td><td>5,81E+05</td><td>tr|A0A068VNV4|A0A068VNV4_COFCA</td><td>Oxidation (M)</td></tr>
<tr><td>WGEDGYIR</td><td>54,96</td><td>994,4508</td><td>8</td><td>-0,5</td><td>498,2325</td><td>14,57</td><td>3,72E+06</td><td>tr|A0A068TZE0|A0A068TZE0_COFCA</td><td></td></tr>
<tr><td>RAGDAPSPGAGSIE</td><td>54,23</td><td>1283,6106</td><td>14</td><td>-1,9</td><td>642,8113</td><td>7,74</td><td>0</td><td>tr|A0A068VNV4|A0A068VNV4_COFCA</td><td></td></tr>
<tr><td>DTGITPDHPSFSDE</td><td>54,11</td><td>1516,6317</td><td>14</td><td>1,8</td><td>759,3245</td><td>15,76</td><td>1,81E+05</td><td>tr|A0A068UNA6|A0A068UNA6_COFCA</td><td></td></tr>
<tr><td>KPDIIGPGVN</td><td>52,96</td><td>1008,5604</td><td>10</td><td>-0,7</td><td>505,2871</td><td>12,81</td><td>3,39E+06</td><td>tr|A0A068UNL8|A0A068UNL8_COFCA:tr|A0A068UNA6|A0A068UNA6_COFCA</td><td></td></tr>
<tr><td>PADDPRNF</td><td>52,52</td><td>930,4195</td><td>8</td><td>-0,5</td><td>466,2168</td><td>15,08</td><td>6,13E+05</td><td>tr|A0A068VNV4|A0A068VNV4_COFCA</td><td></td></tr>
<tr><td>GHVNPPRAID</td><td>52,45</td><td>1074,557</td><td>10</td><td>1</td><td>538,2863</td><td>6,01</td><td>1,88E+05</td><td>tr|A0A068UNL8|A0A068UNL8_COFCA</td><td></td></tr>
<tr><td>NASPGILKPD</td><td>51,11</td><td>1010,5396</td><td>10</td><td>0,1</td><td>506,2771</td><td>11,72</td><td>7,75E+05</td><td>tr|A0A068UNL8|A0A068UNL8_COFCA</td><td></td></tr>
<tr><td>PRQPNGEDM(+15.99)GNFY</td><td>51,06</td><td>1539,6412</td><td>13</td><td>-2,6</td><td>770,8259</td><td>11,05</td><td>3,23E+05</td><td>tr|A0A068VNV4|A0A068VNV4_COFCA</td><td>Oxidation (M)</td></tr>
<tr><td>GPKPGQPLDE</td><td>49,36</td><td>1036,5189</td><td>10</td><td>2,1</td><td>519,2678</td><td>6,87</td><td>3,52E+04</td><td>tr|A0A068UNL8|A0A068UNL8_COFCA</td><td></td></tr>
<tr><td>GAGHVNPPRAID</td><td>48,34</td><td>1202,6156</td><td>12</td><td>2,6</td><td>602,3167</td><td>6,38</td><td>1,94E+05</td><td>tr|A0A068UNL8|A0A068UNL8_COFCA</td><td></td></tr>
<tr><td>AIPHIPIH</td><td>48,19</td><td>896,5232</td><td>8</td><td>-1,5</td><td>449,2682</td><td>14,58</td><td>4,77E+06</td><td>tr|A0A068VNV4|A0A068VNV4_COFCA</td><td></td></tr>
<tr><td>HVNPPRAIDPGLTY</td><td>46,82</td><td>1548,8048</td><td>14</td><td>-0,2</td><td>775,4095</td><td>16,84</td><td>4,77E+05</td><td>tr|A0A068UNL8|A0A068UNL8_COFCA</td><td></td></tr>
<tr><td>ALPADDPRNF</td><td>46,7</td><td>1114,5406</td><td>10</td><td>-0,1</td><td>558,2775</td><td>15,13</td><td>1,49E+07</td><td>tr|A0A068VNV4|A0A068VNV4_COFCA</td><td></td></tr>
<tr><td>VVDQVRVE</td><td>46,4</td><td>942,5134</td><td>8</td><td>0</td><td>472,264</td><td>8,57</td><td>2,44E+05</td><td>tr|D7REL9|D7REL9_COFAR:tr|A0A068VB43|A0A068VB43_COFCA:tr|A0A068V8L2|A0A068V8L2_COFCA</td><td></td></tr>
<tr><td>SIQPVAPADPRVA</td><td>45,46</td><td>1319,7197</td><td>13</td><td>-0,5</td><td>660,8668</td><td>14,7</td><td>7,71E+05</td><td>tr|A0A068VCC7|A0A068VCC7_COFCA</td><td></td></tr>
<tr><td>GITPDHPSFSDE</td><td>44,75</td><td>1300,5571</td><td>12</td><td>-1,4</td><td>651,2849</td><td>14,24</td><td>1,72E+06</td><td>tr|A0A068UNA6|A0A068UNA6_COFCA</td><td></td></tr>
<tr><td>GIIDSGITPGHPS</td><td>44,49</td><td>1249,6302</td><td>13</td><td>-1,6</td><td>625,8214</td><td>16,11</td><td>3,63E+06</td><td>tr|A0A068UNL8|A0A068UNL8_COFCA</td><td></td></tr>
<tr><td>SRGPNNASPGIL</td><td>43,68</td><td>1181,6152</td><td>12</td><td>-1</td><td>591,8143</td><td>13,42</td><td>7,82E+05</td><td>tr|A0A068UNL8|A0A068UNL8_COFCA</td><td></td></tr>
<tr><td>PRQPN(+.98)GEDM(+15.99)GNFY</td><td>42,07</td><td>1540,6252</td><td>13</td><td>-0,6</td><td>771,3195</td><td>12,12</td><td>2,65E+04</td><td>tr|A0A068VNV4|A0A068VNV4_COFCA</td><td>Deamidation (NQ); Oxidation (M)</td></tr>
<tr><td>DLLGPK(+58.01)PGQPLDE</td><td>40,46</td><td>1435,7194</td><td>13</td><td>-1</td><td>718,8662</td><td>22,06</td><td>4,44E+05</td><td>tr|A0A068UNL8|A0A068UNL8_COFCA</td><td>Carboxymethyl (KW, X@N-term)</td></tr>
<tr><td>GGPSK(+58.01)LPFFDDPIAL</td><td>40,02</td><td>1630,8242</td><td>15</td><td>-0,4</td><td>816,4191</td><td>36,85</td><td>1,13E+06</td><td>tr|A0A068UNL8|A0A068UNL8_COFCA</td><td>Carboxymethyl (KW, X@N-term)</td></tr>
<tr><td>KGAVLPVKDQ</td><td>39,79</td><td>1053,6182</td><td>10</td><td>-0,1</td><td>527,8163</td><td>6,25</td><td>7,69E+04</td><td>tr|A0A068TZE0|A0A068TZE0_COFCA</td><td></td></tr>
<tr><td>WVGDPRQPNGE</td><td>38,92</td><td>1253,5789</td><td>11</td><td>0,5</td><td>627,797</td><td>10,31</td><td>2,35E+05</td><td>tr|A0A068VNV4|A0A068VNV4_COFCA</td><td></td></tr>
<tr><td>VNPPRAIDPGLT</td><td>38,89</td><td>1248,6826</td><td>12</td><td>-0,4</td><td>625,3483</td><td>16,56</td><td>3,23E+06</td><td>tr|A0A068UNL8|A0A068UNL8_COFCA</td><td></td></tr>
<tr><td>SSRGPNNASPGIL</td><td>38,69</td><td>1268,6472</td><td>13</td><td>-1,1</td><td>635,3302</td><td>13,24</td><td>1,92E+06</td><td>tr|A0A068UNL8|A0A068UNL8_COFCA</td><td></td></tr>
<tr><td>SSRGPN(+.98)NASPGIL</td><td>37,92</td><td>1269,6313</td><td>13</td><td>-2,3</td><td>635,8215</td><td>14,24</td><td>7,28E+05</td><td>tr|A0A068UNL8|A0A068UNL8_COFCA</td><td>Deamidation (NQ)</td></tr>
<tr><td>GHVNPPRAIDPGL</td><td>37,43</td><td>1341,7153</td><td>13</td><td>4,1</td><td>671,8677</td><td>14,52</td><td>0</td><td>tr|A0A068UNL8|A0A068UNL8_COFCA</td><td></td></tr>
<tr><td>TIDRDIRAT</td><td>37,19</td><td>1059,5673</td><td>9</td><td>-0,5</td><td>530,7906</td><td>5,73</td><td>6,38E+05</td><td>tr|A0A068UNL8|A0A068UNL8_COFCA</td><td></td></tr>
<tr><td>RNDGHIL</td><td>35,67</td><td>823,4301</td><td>7</td><td>-1,1</td><td>412,7219</td><td>6,31</td><td>6,79E+05</td><td>tr|A0A068UNL8|A0A068UNL8_COFCA</td><td></td></tr>
<tr><td>DLLGPKPGQPL</td><td>35,27</td><td>1133,6444</td><td>11</td><td>-0,6</td><td>567,8292</td><td>20,91</td><td>5,87E+06</td><td>tr|A0A068UNL8|A0A068UNL8_COFCA</td><td></td></tr>
<tr><td>GGPSK(+14.02)LPFFDDPIAL</td><td>34,47</td><td>1586,8344</td><td>15</td><td>1,1</td><td>794,4254</td><td>33,48</td><td>2,77E+05</td><td>tr|A0A068UNL8|A0A068UNL8_COFCA</td><td>Methylation(KR)</td></tr>
<tr><td>LGPKPGQPLDE</td><td>34,37</td><td>1149,6029</td><td>11</td><td>-1,4</td><td>575,8079</td><td>12,38</td><td>4,04E+05</td><td>tr|A0A068UNL8|A0A068UNL8_COFCA</td><td></td></tr>
<tr><td>DALPGLTY</td><td>33,07</td><td>848,428</td><td>8</td><td>0,5</td><td>425,2215</td><td>20,22</td><td>1,24E+06</td><td>tr|A0A068VEK9|A0A068VEK9_COFCA</td><td></td></tr>
<tr><td>GGGGAGAGRGPILA</td><td>32,77</td><td>1109,5941</td><td>14</td><td>0,2</td><td>555,8044</td><td>11,35</td><td>1,53E+05</td><td>tr|A0PD76|A0PD76_COFAR:tr|A0A068VDT0|A0A068VDT0_COFCA:tr|A0A068VAJ2|A0A068VAJ2_COFCA</td><td></td></tr>
<tr><td>VYPGM(+15.99)NGDQF</td><td>32,24</td><td>1142,4702</td><td>10</td><td>0,4</td><td>572,2426</td><td>16,49</td><td>7,56E+05</td><td>tr|A0A068UNA6|A0A068UNA6_COFCA</td><td>Oxidation (M)</td></tr>
<tr><td>TGITPDHPSFSDE</td><td>31,82</td><td>1401,6049</td><td>13</td><td>-1,2</td><td>701,8089</td><td>14,84</td><td>7,16E+04</td><td>tr|A0A068UNA6|A0A068UNA6_COFCA</td><td></td></tr>
<tr><td>KLPFFDDPIAL</td><td>31,43</td><td>1274,691</td><td>11</td><td>-0,4</td><td>638,3525</td><td>33,61</td><td>4,76E+06</td><td>tr|A0A068UNL8|A0A068UNL8_COFCA</td><td></td></tr>
<tr><td>GITPGHPSFS</td><td>30,52</td><td>998,4821</td><td>10</td><td>-0,8</td><td>500,2479</td><td>12,72</td><td>7,33E+05</td><td>tr|A0A068UNL8|A0A068UNL8_COFCA</td><td></td></tr>
</table>

KALPADDPRNF	30,36	1242,6356	11	-0,6	622,3247	10,99	4,84E+05	tr|A0A068VNV4|A0A068VNV4_COFCA	
SIQPVAPADPR	29,61	1149,6141	11	0,8	575,8148	10,6	5,80E+05	tr|A0A068VCC7|A0A068VCC7_COFCA	
GGPSK(+72.02)LPFFDDPIAL	28,43	1644,8398	15	-0,2	823,4271	37,03	2,46E+05	tr|A0A068UNL8|A0A068UNL8_COFCA	Carboxyethyl
RWVGDPRQPNGEDM(+15.99)	28,2	1671,7423	14	0,3	558,2549	7,48	4,59E+05	tr|A0A068VNV4|A0A068VNV4_COFCA	Oxidation (M)
NLPVRVAPQ	28,18	992,5767	9	-2,9	497,2942	13,24	1,18E+06	tr|A0A068V471|A0A068V471_COFCA	
SVVNAKF	26,46	763,4228	7	-1,1	382,7183	18,45	1,13E+06	tr|A0A068UQI2|A0A068UQI2_COFCA	
EITGSVHLVG	25,69	1010,5397	10	0	506,2771	11,72	7,75E+05	tr|A0A068V1G4|A0A068V1G4_COFCA	
DSGITPGHPS	24,91	966,4407	10	-2,7	484,2263	6,4	5,27E+04	tr|A0A068UNL8|A0A068UNL8_COFCA	
SAGRDIVFY	24,46	1026,5134	9	-1,8	514,2631	18,84	1,48E+06	tr|A0A068VNV4|A0A068VNV4_COFCA	
VLATHMIL	24,35	896,5154	8	7,3	449,2682	15,33	6,26E+05	tr|A0A068TQZ9|A0A068TQZ9_COFCA	
SIQPVAPADPH(+72.02)VVG	24,22	1457,7513	14	2,8	729,885	16,37	1,35E+05	tr|A0A3P8MYP6|A0A3P8MYP6_COFCA:tr|A0A068VCQ0|A0A068VCQ0_COFCA	Ethoxyformylation
RVLDSNNVVNPPDE	24,04	1566,7638	14	-2	784,3876	13,83	8,80E+04	tr|A0A068UJ50|A0A068UJ50_COFCA	
NAQEPSFRFPS	23,37	1278,5992	11	-0,4	640,3066	19,66	1,56E+06		
PIFAVGPLHK	22,44	1077,6334	10	-4,6	539,8215	19,57	1,20E+06		
LPFFDD(+55.92)PIAL	22,18	1202,5157	10	-0,2	602,265	39,55	7,57E+05	tr|A0A068UNL8|A0A068UNL8_COFCA	
GGPSKLPF	21,8	801,4385	8	-1,7	401,7258	17,43	2,23E+06	tr|A0A068UNL8|A0A068UNL8_COFCA	
FDDPIAL	21,73	789,3908	7	-1,8	395,702	27,73	4,35E+05	tr|A0A068UNL8|A0A068UNL8_COFCA	
ADNAAGRRPGYGVV	21,54	1401,7113	14	-0,7	701,8624	7,78	2,88E+05		
KLEGVSITTY	21,24	1109,5968	10	-2,2	555,8044	11,35	1,53E+05		
RKGTISGSFVP	21,1	1147,6349	11	-0,9	574,8242	17,6	4,80E+05		
LRITDGKSN	21,01	1002,5458	9	3,6	502,282	18,89	2,14E+06		
GPNNASPGILKPD	20,96	1278,6567	13	-3,6	640,3334	12,58	1,66E+05	tr|A0A068UNL8|A0A068UNL8_COFCA	
WVGDPRQPN(+.98)GEDM(+15.99)	20,19	1516,6252	13	-4	759,3169	11,31	0	tr|A0A068VNV4|A0A068VNV4_COFCA	Deamidation (NQ); Oxidation (M)
LPPPPPNPNP	20,07	1038,5498	10	6,4	520,2855	12,36	1,58E+05		
SK(+14.02)ANDPGLVN(+.98)C	19,6	1131,5229	11	-4,2	566,7664	9,64	3,33E+04		Methylation(KR); Deamidation (NQ)
MHGCSSASALIVHA	19,22	1382,6434	14	-1,3	692,3281	14,22	0		
VNRN(+.98)DGHIL	19,03	1037,5254	9	-3,8	519,768	7,77	0	tr|A0A068UNL8|A0A068UNL8_COFCA	Deamidation (NQ)
PAAIKSAIMTTAT	19,01	1274,6904	13	-0,3	638,3523	34,61	4,67E+05		
PAAIKSALMTTAT	19,01	1274,6904	13	-0,3	638,3523	34,61	4,67E+05		
LSTSAAAAATAAVEAAGA	18,93	1502,7576	18	0,3	501,9266	7,26	1,45E+05		
QGAQGPPPML	18,8	994,4906	10	-1,6	498,2518	10,72	2,58E+05		
YCPAVCSY	18,54	904,3459	8	2	453,1811	20,25	5,38E+05		
GYTKGDGLGAEIV	18,48	1278,6455	13	5,2	640,3334	12,58	1,66E+05		
AGRRPGYGVVT	18,32	1131,6149	11	-1,1	566,8141	6,12	9,75E+04		
NGSGGPQF	18,26	762,3297	8	0,5	382,1723	7,04	3,10E+05		
KLPPPPKVN	17,86	988,6069	9	-1,2	495,3102	7,41	7,80E+05		
TSSSLPLVGK	17,77	987,5601	10	-3,2	494,7857	16,53	0		
GEGGGYGKGGGAGVGGGYGGGGEGGG	17,73	1983,8306	26	-2	992,9205	17,95	3,12E+06		
LRAGVGPAVP	17,61	935,5552	10	0,1	468,7849	19,83	6,71E+05		
ADNIRPTDLDM	17,6	1259,5815	11	-5,4	630,7946	8,01	0		
GGYGGGGEAGGGYGKGG	17,45	1356,5693	17	-8	679,2865	10,52	6,82E+04		
PGTSSGAIASPLGPGG	17,38	1324,6622	16	4	442,5631	5,86	0		
RWVGDPRQPNGEDM(+15.99)GNFY	17,35	2152,9385	18	-0,2	718,6533	16,35	1,92E+05	tr|A0A068VNV4|A0A068VNV4_COFCA	Oxidation (M)

LPFFDDPIALGA(+28.03)	17,28	1302,6859	12	5,8	652,354	30,95	1,83E+05	tr|A0A068UNL8|A0A068UNL8_COFCA	Ethylation
DSIATSGTDE	17,12	994,4091	10	2,3	498,213	10,88	7,24E+03		
ETGPAIS	17,11	673,3282	7	-5	337,6697	5,47	0		
NVVPAPAP	16,79	763,4228	8	-0,8	382,7184	20,21	1,80E+06		
LNTSVGVLECK	16,73	1161,6063	11	-0,4	581,8102	37,9	2,42E+06		
EMAYDALKEEG	16,7	1254,5438	11	4,1	628,2817	12,49	3,79E+05		
PSLSTPSASLLG	16,65	1128,6027	12	1,6	565,3095	17,19	5,14E+05		
SVALKNF	16,57	777,4385	7	1	389,7269	18,47	0		
EALLLRPL	16,38	923,5804	8	-1,4	462,7968	23,1	1,08E+06		
VDIPWVK	16,21	855,4854	7	0	428,75	22,27	1,39E+06	tr|A0A068VNV4|A0A068VNV4_COFCA	
NVFATGAGHVN(+15.00)P	15,98	1197,5778	12	-0,2	599,796	6,2	4,87E+05		Deamidation followed by a methylation
EDAVPSPPILLP	15,94	1246,6809	12	-2,2	624,3464	11,52	8,27E+04		
DLLGNGTPR	15,76	941,493	9	-0,7	471,7535	5,73	5,64E+04		
SSPGKGKSGSAWGA	15,54	1275,6207	14	-1,5	638,8167	14,57	9,59E+05		
SIAGGTGSGMGSY	15,47	1143,4866	13	-1,1	572,7499	10,66	8,16E+04		
SGADDRQVK	15,12	974,4781	9	1,5	488,2471	12,5	8,39E+05		
KPITNPPIPA	15,1	1046,6124	10	-1	524,313	10,43	1,91E+05		
